# Numerical Analysis of the Relationship between Nasal Structure and Its Function

**DOI:** 10.1155/2014/581975

**Published:** 2014-02-06

**Authors:** Shen Yu, Xiu-zhen Sun, Ying-xi Liu

**Affiliations:** ^1^State Key Laboratory of Structural Analysis for Industrial Equipment, Dalian University of Technology, Dalian 116023, China; ^2^Otorhinolaryngology Department, Dalian Medical University, Dalian 116023, China

## Abstract

The functions of the nasal cavity are closely related to its structure. In this study the three-dimensional finite element models were established based on the clinical data of twenty-four volunteers to study the influence of nasal structure on nasal functions of heating the inhaled airflow. Numerical simulations mainly concerning the airflow distribution and the airflow temperature are performed. The character of airflow heating process in these models is gained from the simulation results of these nasal cavities. The parameters describing the geometry of nasal cavity, such as the surface area of nasal airway and the volume of nasal cavity, are considered to be related to the nasal function of heating the inhaled airflow. The approximate function describing the relationship between the geometric parameters of the nasal airway and the nasal functions is gotten. This study can provide a numerical platform for studying some clinical problems and will contribute to the further research on the relationship between nasal structure and nasal functions.

## 1. Introduction

The nose acts as an air-conditioning device, which primarily performs the functions of heating, humidifying, and removing particulate and gaseous pollutants from inspired air before it approaches the lung. The airflow distribution and the nasal functions will be affected by structural changes of the nasal airway initiated by nasal diseases [1, 2]. It is necessary to explore the complex form of how the nasal structure affects the nose accomplishing its disparate functions. Keck et al. [3] measured temperature of 23 volunteers with a miniaturized thermocouple sensor during respiration at different locations in the nasal cavity. The temperature of the anterior nasal segment airflow was found highly increased during the inspiration, the increased value of which in the posterior segment was less prominent in spite of the longer distance. Lindemann et al. [4] studied the influence of surgical closure of septal perforations on intranasal temperature to evaluate changes in clinical symptoms after surgery. It was found that the increase in the value of temperature at the anterior turbinate area was significantly higher postoperatively. Recently, computational models of nasal transport characteristics were developed to compensate for the limited *in vivo* data due to the inaccessibility of the nasal cavity. Naftali et al. [5] built three three-dimensional nasal models including two simplified models and one anatomical accurate model to study air transport patterns in the human nose and its overall air-conditioning capacity. The study suggested that a healthy nose can efficiently provide about 90% of the heat and the water fluxes required to condition the ambient inspired air to near alveolar conditions in a variety of environmental conditions. The anatomical replica of the human nose showed the best performance. Keck et al. reported the influence of the nasal structural changes due to nasal surgery on the nasal capacity [6, 7].

The objective of this research is to investigate the influence of nasal cavity structure on nasal functions such as heating inspired air. Simultaneously, considering individual variations in nasal structure, twenty-six three-dimensional nasal models are established including twenty-four normal nasal cavities and two variants with partial removals of the turbinate in a selected nasal cavity. Numerical simulations for inspiratory airflow are performed using the finite element method under steady-state conditions. Simulation results are analyzed to describe the relationship between the nasal functions and the nasal structure geometry.

## 2. Modeling and Computations

**Figure 1:**
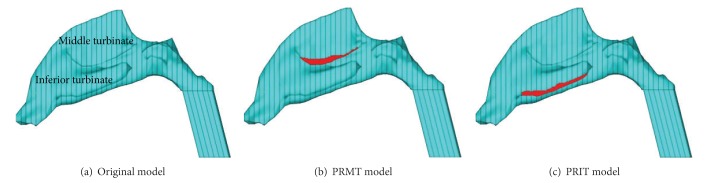
The selected nasal model (a) and the two variants ((b), (c)). Model (a) is the nasal model. Model (b) is the variant model with partial middle turbinate-removed (PRMT). Model (c) is the variant model with partial inferior turbinate-removed (PRIT). The red area shows the part which was removed.

**Figure 2:**
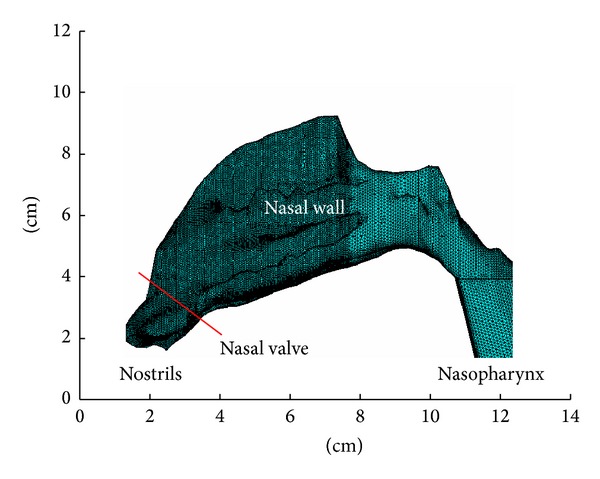
The three-dimensional finite element model of a nasal cavity.

The clinical data of twenty-four volunteers are used in this study. All of the subjects are volunteers. We have explained the purpose of the test to the volunteers and they all agreed with it. With a spatial resolution of 512∗512 pixels, about 120 coronal slices, and 1 mm thickness, the CT data (DICOM format images) of these volunteers' nasal cavity are acquired for the reconstruction. Based on the 1 mm CT scan images, twenty-four three-dimensional nasal models were established. A nasal model was selected and partial left inferior and middle turbinates were removed, respectively, to form two variant models. The selected model and its two variants were used to describe the influence of nasal structure change on the nasal heating function. As shown in Figure 1, the middle photo (b) and the right photo (c) show, respectively, the models with partial removal of middle turbinate (PRMT) and the model with partial removal of inferior turbinate (PRIT). The red area shows the part which was removed. These twenty-six numerical models were built and meshed with tetrahedral elements using the software of ANSYS as shown in Figure 2. The meshing quality of the tetrahedral elements was good without any distorted elements.

Steady-state inspiratory air and heat transport were simulated using the finite element method (ANSYS). Concerning the boundary conditions, a no-slip flow velocity on the passage surfaces was assumed. At the nostril, a pressure condition (*P* = 101.325 kPa) was specified which equals a standard atmospheric pressure. At the nasopharynx, a velocity condition was specified which was acquired from the volumetric flow of 600 mL/s and the cross-sectional area of nasopharynx. The volumetric flow of 600 mL/s, which determined the flow regime to be turbulent [8–10], is a high but reasonable value regarded as a Chinese respiration flow rate value [11]. The *k*-*ε* turbulence model was used to simulate the turbulence flow in the nasal cavities [2].

Lindemann et al. [12] measured mucosa temperature at several intranasal sites in 15 healthy volunteers at room conditions (25 ± 1°C and 30 ± 4% relative humidity). Based on the measurement of Lindemann and the report of Garcia, the boundary conditions of temperature were defined for the simulation of heat transport in nasal cavities. The temperature of ambient air was set to 25°C at the nostrils. The values of 32.6°C for the mucosal temperature of proper nasal cavity and 34.4°C for the mucosal temperature of nasopharynx were defined during inspiration. The measurement of Lindemann showed that the temperature values in the nasal valve area and the anterior turbinate area were significantly lower than in the nasal vestibule at the end of inspiration and expiration. Thus an average value of 33.5°C was adopted for the nasal vestibule.

## 3. Results

**Figure 3:**
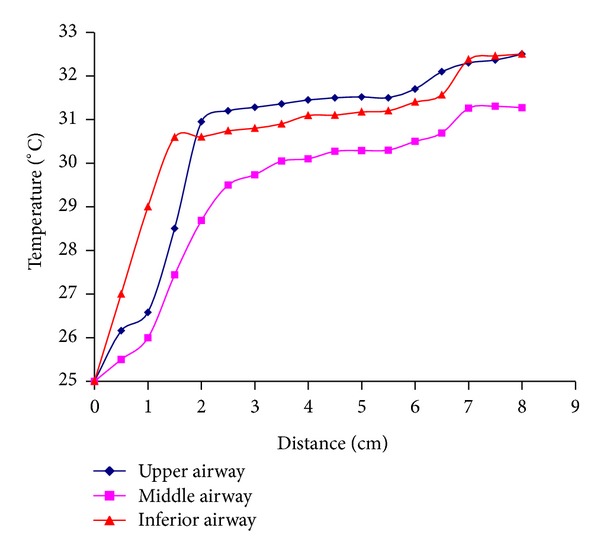
Different heating streamlines of airflow in three nasal airways of a selected nose. The blue, purple, and red lines show, respectively, the heating effect of air streamline in the upper airway, middle airway, and inferior airway.

Numerical simulations for the transport of heat in these twenty-six nasal models were performed using the commercial software ANSYS. The temperature distribution in the nasal cavities was simulated and analyzed. The heating efficiency from the nostril to the end of the septum was the focus of the study. From the simulation results, it can be investigated that the inspired air was heated mainly in the anterior portion of the cavity. Three streamlines were chosen in the top, middle, and bottom of one cavity to show the function of heating air in the corresponding portion. As shown in Figure 3, temperature increased rapidly in the anterior portion of the nasal cavity and the increasing rate reduced gradually in the posterior portion. The heating function was more effective in the top and bottom of the nasal passages than that in the middle passage. A nasal cavity was selected to define the boundary conditions of different flow rates of 120 mL/s, 240 mL/s, and 600 mL/s in the nasopharynx. By being heated in the nasal cavity, the temperature of air flow was increased to 31.8°C, 30.3°C, and 29.7°C in the left cavity and 32.3°C, 31.4°C, and 30.3°C in the right cavity. It can be concluded that lower flow rates of inspired air are associated with more complete heating.

**Figure 4:**
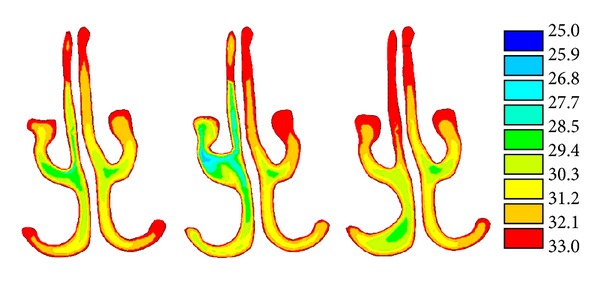
Airflow temperature distribution in the selected nasal model and its two variants.

From Figure 4 it can be seen that the airflow temperature distribution in the selected nasal model was different from that in the two variant models. The airflow temperature was lower in middle airway of the PRMT model and inferior airway of the PRIT where the airway became wider than that of the original model. Partial resection of nasal turbinates resulted in the airflow redistribution and the airflow temperature distribution also changed in the two variant nasal models. The simulation results of the partial left nasal turbinate-removed model are shown in Table 1. The temperature exhibited slightly different value at the end of three cavities. It can be concluded that overall air-conditioning capacity of the nose is almost unaffected by local geometrical variations of the turbinates.

**Figure 5:**
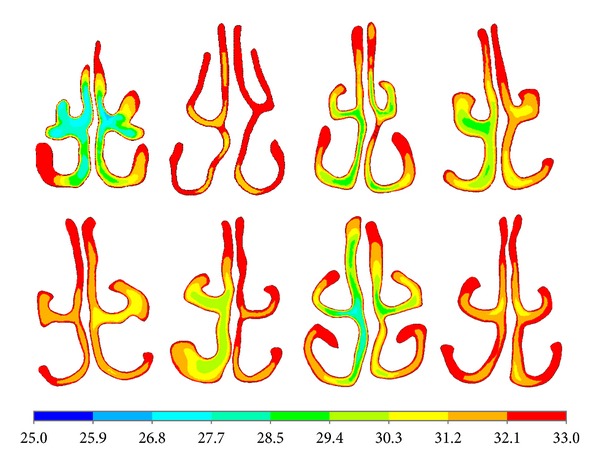
Airflow temperature in different size airway.

Figure 5 showed the temperature distribution in eight nasal cavities. From Figure 5 it can be seen that the airflow temperature distributions were different in every nasal model. The general rule was that the wider the nasal airway was, the lower the airflow temperature was.

**Figure 6:**
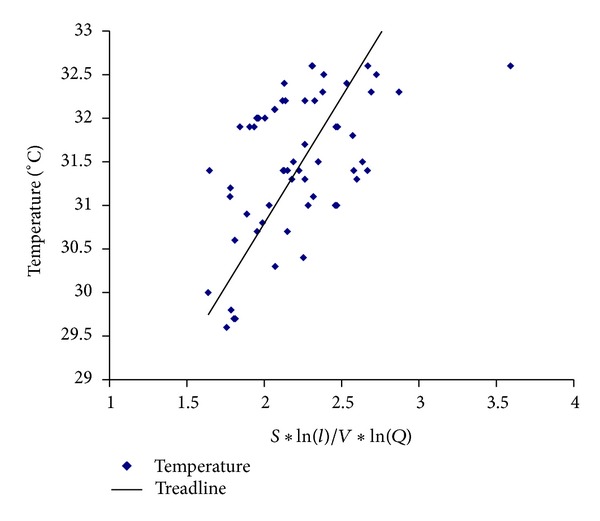
The heating function (left) versus nasal structure.

From the above results it can be deduced that the nasal capacity of heating airflow was related to the nasal cavity geometry and the airflow flux. The capacity of airflow heating was direct proportion with the surface area and length of nasal cavity but inverse proportion with the volume of nasal cavity and airflow flux. Twenty-six noses and fifty-two cavities in all were studied to found the relationship between nasal geometry and nasal capacity. A parameter *f* = (*S*∗log⁡(*l*)/*V*∗log⁡(*Q*)) was applied to express the effect of nasal geometry and airflow flux where *S* and *l* are the surface area and length of nasal cavity and *V* and *Q* are the volume of nasal cavity and airflow flux. The temperature difference (Δ*T*) between nostril and postnaris was used to represent the nasal capacity. As shown in Figure 6, when the parameter is smaller than 3 the temperature of airflow changed linearly with the parameter. When the parameter is greater than 3 the temperature of airflow hardly changed with the parameter.

The fitting curve is summarized from the data points in Figure 6. It can be considered linear relationship approximately between parameter *f* and temperature when 1.5 < *f* < 3, shown as

(1) $$\begin{array}{c} \Delta T=\alpha \times f\;1.5<f<3, \end{array}$$

where *α* are undetermined coefficients. The temperature was almost the same as nasal wall when *f* > 3. So the data (*f* > 3) was excluded and the undetermined coefficients were determined as *α* = 2.91. The correlation coefficient *R* was 0.51, respectively, related to the two fitting curves.

## 4. Discussion

The temperature of inhaled air under normal condition was lower than that of nasal mucous. When the airflow passed through nasal cavities the transportation of heat is inevitable. The results show that the exchange of heat occurred mainly in the anterior portion of the cavity in the healthy noses (Figure 3). And it was in the anterior portion of the cavity that the significant differences of temperature between mucosal surface and entering air exist and cause high heat flux. After the air passed through the anterior portion of the cavity the differences of temperature between mucosal surface and airflow became smaller. The exchange of heat in per unit area reduced in the posterior portion of the nasal wall, whose trend is consistent with the study by Garcia et al. [13]. Keck et al. [3] measured the intranasal temperature of 23 volunteers during respiration at different locations in the nasal cavity. The end-inspiratory temperature data was obtained with a miniaturized thermocouple. In his measurement the air temperature is 25 ± 2.1°C in the nasal vestibule, 28.9 ± 3.1°C in the nasal valve, and 32.6 ± 1.5°C in the nasopharynx at the end of inspiration, which were close to the average data of this study (25°C 29.2°C 31.9°C), respectively. The temperature of all volunteers also shows that the anterior part of nasal cavity is the main area of heat exchange [3, 4]. The boundary conditions for numerical simulation and the actual condition cannot be exactly the same, which may cause some difference between the computational results and the test results. In the similar conditions the above comparisons show that the simulation results in this study are relatively reliable.

From the simulation results of partial removed nasal turbinate model (Table 1), it was obtained that local variations in the structural proper nasal cavity would not change the volume and area of nasal cavity significantly so that the air-conditioning capacity of the nose was deemed to be practically unaffected. These results were in agreement with the study by Naftali et al. [5] and Elad et al. [7]. The airflow flux also affected the air-conditioning capacity of the nose. If the airflow flux was small the airflow would have a low average velocity and have enough time to be heated. As shown in Figure 3 the heating effect of air streamline was better in the top and bottom of the nasal passages than that in the middle passage just because most of airflow (about 59%) passed through the middle passages and a small part of airflow passed through the top (about 8%) and bottom (about 33%) passages [2]. The airflow had enough time to be heated and the temperature was more closed to that of the nasal wall in the top and bottom passages.

As shown in Figure 6 the temperature of airflow was related to the structural geometry parameter (*S*∗log⁡(*l*)/(*V*∗log⁡(*Q*))) of the nasal cavity. *S* was the nasal surface area, *V* was the nasal cavity volume, *l* was the length between anterior naris and posterior naris, and *Q* was the airflow flux. When the *V* was constant and the *S* was larger, the effect of heating was better. The larger the ratio of *S*/*V* was, the narrower the nasal cavity was, the nasal resistance higher was, and vice versa [13]. Lindemann et al. [12] reported that the smaller the nasal resistance was, the higher the temperature of nasal mucous was. It was indicated that the nasal mucous transported less heat to the inhaled air and the heating effect decreased. A large *l* can increase the time spent of passing through the nasal cavity so that it can make the airflow heated sufficiently. High increase in air temperature coming along with low airflow velocities and the wide nasal airway can reduce blending of the air adjacent to the nasal wall with the air in the centre causing decreased heating of the inspiratory air [12]. These two sentences want to express the meaning that when airflow velocity was low the air temperature would increased rapidly, and when the nasal airway was wide the air temperature would increased slowly. This means that the temperature increase is inversely proportional to the airflow rate and the volume of the nasal cavity. He also thought that the determining factors influencing the air temperature distribution were the nasal wall surface area. From the above discussion we can conclude that the larger the geometry parameter (*S*∗log⁡(*l*)/(*V*∗log⁡(*Q*))) was, the better the nasal heating effect was. When the parameter value was great than 3, the temperature of inhaled airflow in the naropharynx was close to the nasal mucous and the temperature would not increase with the geometry parameter anymore. The nasal cavity was so narrow that it restricts the normal breath with the parameter value great than 3. During nasal surgery the nasal reconstruction should be done with careful consideration of postsurgical geometry. The width of nasal passages improved by operation should be appropriate because wide passages have undue influence on the conditioning function of inhaled airflow and narrow passages can affect normal breath. The moderate reduction of “*f*” value was conductive to the breath function, and the nose can still perform its conditioning functions on the inhaled airflow. The parameter “*f*” is a reference for surgical planning which clarifies the influence of each nasal structure parameter on the nasal functions.

We can also draw a conclusion from Figure 6 that the data points dispersed in some extent mainly because the effect of heating was related to the specific geometry of nasal cavity except length, surface area, and volume of nose. Additionally, the anterior part of nose was the major heating area for inhaled airflow from which the geometry parameter should be considered to be selected. All the reasons mentioned above are to some extent contributing to the deviation of result data. Equation (1) was an approximate expression.

## 5. Conclusion

Simulations for heating airflow were performed within twenty-six CFD nasal models to study the influence of nasal cavity structure on nasal function of heating the inhaled airflow. The anterior part of nose was the main heating area for inhaled airflow. The parameters of surface area, length, volume of nasal cavity, and airflow flux would affect the performance of nasal functions. The selected geometry parameter “*f*” can reflect the nasal functions in some extent. This study can provide some reference data for clinical problem and will contribute to the further research on the influence of nasal cavity structure on nasal function.

## Figures and Tables

**Table 1:** Simulation results of heating airflow in the selected nasal model and its two variants.

	*T* (Δ*T*) (°C)		Area (cm^2^)		Volume (cm^3^)	
	Left cavity	Right cavity	Left cavity	Right cavity	Left cavity	Right cavity
Original model	30.8 (5.8)	30.6 (5.6)	75.5	75.3	11.9	12.7
Model of PRMT	30.4 (5.4)	30.9 (5.9)	74.2	75.3	12.2	12.7
Model of PRIT	30.6 (5.6)	30.7 (5.7)	73.8	75.3	12.7	12.7
